# 2.H. Workshop: Commercial determinants and global capture of public health- cases spanning the world

**DOI:** 10.1093/eurpub/ckac129.087

**Published:** 2022-10-25

**Authors:** Organised by: Governance, Ethics and Conflict of Interest in Public Health Network, UK Faculty of Public Health, Chair persons: Jihad Makhoul (Lebanon)

## Abstract

Growing research around the globe shows that for-profit corporations incur increasingly adverse impacts on health and well-being of people, the planet and the global economy. Their health damaging products and practices fuel non-communicable disease epidemics, damage the earth's natural environment and interfere in health policy making. Such corporate interference in social and public health policy, research and practice is well documented for the pharmaceutical, tobacco, food and beverage, alcohol and arms industries. A commercial determinant lens allows a better understanding of heath inequalities by drawing attention to corporate actors and their tactics as drivers of ill-health rather than people's behaviors. This framework also makes it possible to present interventions to counter these influences. This workshop panel of 5 scholars and activists from around the world who are members of the Governance, Ethics and Conflict of Interest in Public Health Network serves to confirm the detrimental reach of industry practices and the global responses to this interference in public health. It aims to add to the emerging body of knowledge on commercial determinants of health using innovative research approaches and findings. The format is a series of 5 short sequential presentations, followed by an interactive discussion with the audience, moderated by the panel organizer. The presentations will showcase the research methods used including multi-stakeholder interviews, policy analyses, systems mapping and analyses of policy debates. The presentations will also document examples and cases of corporate capture of food industry in Europe, a global mapping of corporate systems, industry interference in health-protecting laws in Columbia, exploitation of humanitarian emergencies in Lebanon, and suggestions of ways forward to protect adults and children from industry vested interests with a Public Health Playbook.

**Key messages:**

• Corporate interference in public health is a growing concern globally for its health harming influence on health systems and future generations.

• Continued concerted research revealing overt and covert health harming industry practices is needed to counter their impact on PH in a world fraught with global challenges and uncertainty.

